# Facilitadores, barreiras e estratégias para ampliar o uso dos marcadores do consumo alimentar na atenção primária à saúde

**DOI:** 10.1590/0102-311XPT119224

**Published:** 2025-02-07

**Authors:** Joanna Manzano Strabeli Ricci, Agatha Cosmo de Moura Balbino, Pedro Henrique Benicio Oliveira, Priscila de Morais Sato, Bárbara Hatzlhoffer Lourenço

**Affiliations:** 1 Faculdade de Saúde Pública, Universidade de São Paulo, São Paulo, Brasil.; 2 Escola de Nutrição, Universidade Federal da Bahia, Salvador, Brasil.

**Keywords:** Vigilância Alimentar e Nutricional, Sistemas de Informação em Saúde, Grupos Focais, Pesquisa Qualitativa, Food and Nutritional Surveillance, Health Information Systems, Focus Groups, Qualitative Research, Vigilancia Alimentaria y Nutricional, Sistemas de Información en Salud, Grupos Focales, Investigación Cualitativa

## Abstract

Este estudo buscou compreender facilitadores, barreiras e estratégias para o uso de marcadores do consumo alimentar por profissionais da atenção primária à saúde (APS). As respostas de 235 profissionais de saúde a um questionário eletrônico foram utilizadas para desenvolver um roteiro para grupos focais realizados virtualmente. Em 10 grupos focais, 34 profissionais de cargos assistenciais e gestão de todas as macrorregiões brasileiras foram incluídos e as transcrições das gravações foram analisadas tematicamente. Profissionais da assistência e gestores enfatizaram a necessidade de infraestrutura para o uso adequado dos marcadores, apontando falta de equipamentos e instabilidade na conexão como principais barreiras. Gestores destacaram a possibilidade de uso por qualquer profissional como facilitadora, ao passo que profissionais da assistência assinalaram positivamente a estrutura dos marcadores, mas a falta de sensibilização sobre sua importância foi mencionada como barreira pelos participantes. Apesar da lentidão, a acessibilidade das plataformas para registro dos marcadores foi apontada como facilidade, assim como a condicionalidade com políticas públicas, especialmente quando vinculadas a incentivos financeiros. Os grupos focais permitiram trocas de estratégias, como educação permanente, treinamentos para digitadores, formas de uso dos marcadores nos espaços de assistência à saúde e ampliação da divulgação dos dados. Investir em infraestrutura, qualificação profissional e colaboração entre equipes da APS, com compartilhamento de estratégias, foram fatores cruciais para fortalecer o uso dos marcadores do consumo alimentar.

## Abstract (en)

This study aimed to understand the facilitators, barriers, and strategies for the use of food intake markers by primary health care (PHC) professionals. The responses of 235 healthcare health care professionals to an electronic questionnaire were used to develop a script for virtually conducted focus groups. In 10 focus groups, 34 professionals from healthcare and management positions from all Brazilian macroregions were included, and the transcripts were thematically analyzed. Healthcare professionals and managers emphasized the need for infrastructure to properly use the markers, pointing to the lack of equipment and connection instabilities as the main barriers. Managers highlighted the possibility of its use by any professional as a facilitator, whereas healthcare professionals positively indicated the structure of the markers. However, participants mentioned the lack of awareness about their importance as a barrier. Despite its slowness, the accessibility of the platforms to register the markers emerged as a facilitator, as did its conditionality with public policies, especially when linked to financial incentives. The focus groups allowed the exchange of strategies, such as continuing education, training for typists, ways to use markers in health care spaces, and expansion of data dissemination. Investment in infrastructure, professional training, and collaboration between PHC teams, with the sharing of strategies, configured crucial factors to strengthen the use of food intake markers.

## Resumen

Este estudio pretende identificar los facilitadores, las barreras y las estrategias para el uso de marcadores de consumo de alimentos por parte de los profesionales de la atención primaria de salud (APS). Las respuestas de 235 profesionales de la salud a un cuestionario electrónico se utilizaron para desarrollar un guion para los grupos focales que se realizaron virtualmente. En 10 grupos focales, se incluyeron a 34 profesionales de puestos de atención y gestión de todas las macrorregiones brasileñas, y las transcripciones se analizaron temáticamente. Los profesionales y gestores de la asistencia enfatizaron la necesidad de una infraestructura para el uso adecuado de los marcadores, señalando como principales barreras la falta de equipos y la inestabilidad en la conexión. Los gerentes destacaron la posibilidad de uso por parte de cualquier profesional como facilitador, mientras que los profesionales de la atención señalaron positivamente la estructura de los marcadores, pero la falta de conciencia sobre su importancia fue mencionada como una barrera por los participantes. A pesar de la lentitud, se señaló como fácil la accesibilidad a las plataformas de registro de los marcadores, así como la condicionalidad con las políticas públicas, especialmente cuando se vinculan a incentivos financieros. Los grupos focales permitieron intercambiar estrategias, como la educación permanente, la capacitación de digitadores, las formas de uso de marcadores en los espacios de atención a la salud y la ampliación de la difusión de datos. La inversión en infraestructura, la calificación profesional y la colaboración entre los equipos de APS, con el intercambio de estrategias, fueron factores cruciales para fortalecer el uso de marcadores de consumo de alimentos.

## Introdução

A má nutrição associa-se consistentemente à mortalidade e à perda de anos de vida por incapacidade globalmente ^1^ ^,^ ^2^. No Brasil, entre 2002 e 2019, houve um aumento maior que o dobro na ocorrência de obesidade entre homens (de 9,6% para 22,8%) e mulheres (14,5% para 30,2%) ^3^, acompanhada do aumento nas prevalências de adultos com diagnóstico de diabetes mellitus e hipertensão ^4^. Abordagens abrangentes no sistema de saúde para lidar com os desafios alimentares, nutricionais e de saúde pública mostram-se essenciais.

No Sistema Único de Saúde (SUS), ações de vigilância alimentar e nutricional na atenção primária à saúde (APS) incluem a avaliação de marcadores do consumo alimentar ^5^, que constituem um método curto de avaliação da dieta. Como ferramenta de triagem, são deliberadamente simplificados e adequam-se à rotina e à constituição de equipes da APS. Refletindo diretrizes do *Guia Alimentar para a População Brasileira*, os marcadores mensuram aspectos importantes da qualidade global da alimentação ^6^ ^,^ ^7^, que se relacionam à problemática de excesso de peso e doenças crônicas.

Os registros de consumo alimentar são inseridos na plataforma e-SUS APS (https://sisaps.saude.gov.br/esus/), por meio dos softwares Prontuário Eletrônico do Cidadão (PEC) ou Coleta de Dados Simplificada (CDS). Esses dados migram para o Sistema de Informação em Saúde para Atenção Básica (SISAB) e são consolidados no Sistema de Vigilância Alimentar e Nutricional (SISVAN). No SISVAN, os dados podem ser consultados em formato de relatórios, a fim de subsidiar o planejamento da atenção nutricional e de ações para promoção da alimentação adequada e saudável, bem como a prevenção e o monitoramento de agravos à saúde ^5^ ^,^ ^8^. Entretanto, em 2019, a cobertura populacional média do registro de marcadores no Brasil foi de apenas 0,92%, evidenciando a subutilização ante seu potencial, ainda que as tendências sejam ascendentes desde 2015 ^9^.

Investigações anteriores sobre potencialidades e desafios para uso do SISVAN em municípios de Minas Gerais não tiveram foco nos marcadores do consumo alimentar ^10^ ^,^ ^11^ ^,^ ^12^. Rolim et al. ^10^ conduziram estudo transversal com questionário estruturado eletrônico direcionado aos responsáveis municipais do SISVAN, sem abranger a perspectiva dos profissionais da assistência na APS, os quais desempenham papel crucial na execução da vigilância alimentar e nutricional. Alves et al. ^11^, por sua vez, realizaram estudo exploratório de caráter descritivo utilizando entrevistas individuais semiestruturadas com profissionais da assistência, mas essa abordagem não proporcionou a interação entre os participantes.

Para elucidar questões da rotina dos serviços relevantes ao emprego de marcadores do consumo alimentar é fundamental ouvir profissionais em posições de assistência à saúde e gestão. A partir de uma abordagem metodológica que favorece discussões mais aprofundadas sob múltiplas perspectivas, este estudo objetivou compreender as percepções destes profissionais em relação a facilitadores, barreiras e estratégias para a utilização dos marcadores do consumo alimentar no SUS.

## Metodologia

### Desenho do estudo

Esta pesquisa investigou percepções de profissionais da assistência e gestores da APS sobre o uso dos marcadores do consumo alimentar do SISVAN utilizando abordagem qualitativa com delineamento em duas camadas. Inicialmente, um questionário eletrônico levantou facilitadores e barreiras no uso dessas ferramentas. As respostas subsidiaram a elaboração de um roteiro para grupos focais, conduzidos para aprofundar compreensões com a interação entre participantes.

### Participantes

Profissionais e gestores envolvidos no uso dos marcadores do consumo alimentar na APS foram incluídos no estudo por meio da técnica de amostragem bola de neve ^13^. Ou seja, foram inicialmente convidados a responder o questionário e em seguida, convidados a participar dos grupos focais e incentivados a indicarem outros possíveis participantes.

### Construção dos dados

Foi elaborado um questionário para distribuição eletrônica a profissionais e gestores da APS, via Google Formulário (https://www.google.com/forms/) Com questões objetivas, foram abordadas características dos participantes (sexo, escolaridade, profissão, cargo) e fluxo do uso dos marcadores do consumo alimentar nos serviços (onde, por quem e como são utilizados). Percepções sobre barreiras e facilitadores para o uso foram captadas por questões discursivas. O questionário foi divulgado entre julho e outubro de 2021 com apoio da instituição sede da pesquisa, da Coordenação-Geral de Alimentação e Nutrição do Ministério da Saúde e de Conselhos Regionais de Nutricionistas (CRN 1 ₋ Goiás, Mato Grosso, Tocantins e Distrito Federal; CRN 3 ₋ Mato Grosso do Sul e São Paulo; CRN 8 ₋ Paraná; e CRN 9 ₋ Minas Gerais) via e-mails, redes sociais e grupos de WhatsApp.

Respostas ao questionário embasaram a elaboração de um roteiro estruturado para condução de grupos focais. Um grupo focal piloto ^14^ ^,^ ^15^ realizado com profissionais e gestores avaliou a pertinência e fluidez do roteiro, com ajustes posteriores pelos pesquisadores. Ao final, o roteiro abrangeu questões sobre: (i) coleta, inserção no sistema e análise de dados do consumo alimentar; (ii) facilitadores, barreiras e estratégias para uso dos marcadores; (iii) recursos necessários; e (iv) receptividade dos usuários (Quadro 1).

**Quadro 1:** Roteiro de perguntas para grupos focais sobre o uso dos formulários de consumo alimentar na atenção primária à saúde (APS).

TEMA	PERGUNTA
Aplicação (coleta, inserção no sistema e análise dos dados)	Como os formulários de consumo alimentar se relacionam com a promoção da saúde na opinião de vocês?
	Como o preenchimento dos formulários de consumo alimentar é inserido na rotina dos serviços de saúde?
	Qual é a percepção de vocês sobre as plataformas Sisvan Web e e-SUS APS (PEC/CDS)?
	O que é feito a partir das análises dos dados dos formulários?
Dificuldades e facilidades	Quais são as maiores dificuldades que vocês encontram para o preenchimento dos formulários de consumo alimentar?
	E quais são as principais facilidades que vocês encontram para preencher os formulários de consumo alimentar nos serviços de saúde?
	Com base nas experiências de vocês, que estratégias foram utilizadas para aumentar o uso dos formulários de marcadores de consumo alimentar?
	Na opinião de vocês, para que servem os formulários de marcadores de consumo alimentar?
Recursos físicos	Já que falamos dos recursos profissionais, vamos agora falar dos demais recursos: físico, financeiro, tempo, gestão... Na sua UBS, o que tem e o que falta para implementar a utilização dos formulários de marcadores de consumo alimentar? Me contem um pouco sobre isso.
Receptividades dos usuários	E com relação aos usuários, como vocês percebem que as perguntas dos formulários de consumo alimentar são compreendidas?
Desafios de contextos específicos	Agora com relação à situação da COVID-19 que estamos vivendo atualmente. Como vocês sentem que a pandemia pode ter afetado na utilização dos formulários de marcadores de consumo alimentar nos serviços de saúde?

CDS: Coleta de Dados Simplificada; PEC: Prontuário Eletrônico do Cidadão; SISVAN: Sistema de Vigilância Alimentar e Nutricional; SUS: Sistema Único de Saude; UBS: unidade básica de saúde.

Fonte: elaboração própria.

Os grupos foram realizados virtualmente, entre julho e outubro de 2022, pela plataforma Google Meet (https://meet.google.com/) devido suas facilidades para uso (dispensa *download* ou inscrição prévia) e ferramentas disponíveis (*chat* síncrono e gravação de áudio e vídeo). Profissionais e gestores que previamente responderam ao questionário eletrônico foram convidados e encorajados a indicar outros possíveis participantes. Dada a dinâmica de baixas taxas de resposta e comparecimento documentada na literatura ^16^ e observada neste estudo, aproximadamente 30 participantes foram convidados para cada grupo focal.

Os grupos foram programados para uma duração de 45 a 90 minutos e contaram com a presença de uma moderadora (A.C.M.B.), responsável por realizar as questões e manter o foco da conversa, e um observador (P.H.B.O.), que fez anotações durante as falas, como gestos e expressões faciais. Todos os grupos foram gravados e os áudios dessas gravações foram transcritos na íntegra por uma empresa especializada. As anotações do observador foram inseridas ao longo das transcrições, contribuindo para avaliação de reações, concordâncias ou discordâncias. Foi utilizado o critério de saturação ^17^ para definir o número de grupos focais suficientes para esgotar os temas discutidos pelos participantes.

### Análise dos dados

As respostas do questionário eletrônico foram organizadas e categorizadas por uma pesquisadora (J.M.S.R.) da seguinte maneira: (i) pré-análise com leitura flutuante do *corpus* para identificação dos tópicos principais; (ii) sinalização de respostas que apresentavam ideias semelhantes; (iii) releitura e reagrupamento de respostas sinalizadas, com consequente surgimento e nomeação de subcategorias de acordo com as ideias nelas contidas; e (iv) leitura final do *corpus* e ajustes, quando necessário, a categorias e subcategorias segundo relevância e semelhança de temas.

As transcrições dos grupos focais passaram por análise de conteúdo temática de caráter indutivo, com temas emergentes, conforme proposto por Bradley et al. ^18^: (i) leitura imersiva dos textos para compreensão de significados em sua totalidade, com identificação de temas emergentes; (ii) leitura conjunta para consenso entre autores sobre temas e códigos; (iii) elaboração do livro de códigos, com descrição de sentido, critérios de inclusão e exclusão, exemplos típicos, atípicos e “quase, mas não”; (iv) aplicação e discussão do livro de códigos sobre parte do material por dois pesquisadores independentemente (J.M.S.R. e P.H.B.O.), com concordância satisfatória (kappa = 0,87 e > 80% de sobreposição entre trechos) ^19^; (v) aplicação do livro de códigos no material restante (J.M.S.R.); e (vi) interpretação e narração dos sentidos em cada código. A análise de conteúdo foi realizada no MAXQDA Plus 2022, versão 22.6.1 (https://www.maxqda.com/). Para identificar discrepâncias entre percepções de profissionais e gestores, os códigos foram quantificados, com recurso automático do software, levando em consideração a categoria com o cargo do orador do trecho codificado.

### Aspectos éticos

Esta pesquisa foi aprovada pelo Comitê de Ética em Pesquisa da Faculdade de Saúde Pública da Universidade de São Paulo (parecer nº 4.826.601). A construção de dados foi realizada após assinatura do Termo de Consentimento Livre e Esclarecido.

## Resultados

Entre profissionais e gestores que responderam ao questionário (n = 235) e participaram dos grupos focais (n = 34), a maioria era do sexo feminino, entre 30 e 39 anos e possuiam pós-graduação completa. Predominaram profissionais da saúde, especialmente nutricionistas e enfermeiros, com representantes de todas as macrorregiões brasileiras, destacando-se o Sudeste (Tabela 1).

**Tabela 1:** Distribuição dos profissionais da atenção primária à saúde participantes por sexo, idade, escolaridade e profissão.

Características sociodemográficas	Questionário eletrônico (n = 235)		Grupos focais (n = 34)		Total (n = 469)
	n	%	n	%	n
Sexo					
Feminino	214	91	32	94	246
Masculino	21	9	2	6	23
Idade (anos) *					
≤ 29	44	19	2	6	46
30-39	112	48	15	44	127
40-49	47	20	8	24	55
≥ 50	30	13	7	21	37
Escolaridade					
Até Ensino Médio	14	6	1	3	15
Ensino Superior incompleto	6	3	0	0	6
Ensino Superior completo	61	26	6	18	67
Pós-graduação	154	66	27	79	181
Profissão					
Agente comunitário de saúde	9	4	1	3	10
Enfermeiro	78	33	4	12	82
Nutricionista	123	52	29	85	152
Outros **	16	7	0	0	16
Cargo					
Assistência	149	63	17	50	166
Gestão	86	37	17	50	103
Macrorregião					
Centro-oeste	18	8	1	3	19
Nordeste	71	30	7	21	78
Norte	24	10	7	21	31
Sudeste	96	41	14	41	110
Sul	26	11	5	15	31

Fonte: elaboração própria.

* A soma não reflete o número total de respostas devido a dois valores ausentes resultantes de inconsistências nas datas de nascimento;

** Outras profissões: assistente social, auxiliar administrativo, digitador, fisioterapeuta, motorista, psicólogo e terapeuta ocupacional.

O Quadro 2 destaca categorias e subcategorias sobre facilitadores e barreiras na utilização dos marcadores do consumo alimentar apuradas no questionário. Facilidades incluíram objetividade no uso, interface dos sistemas de informação e aplicabilidade por diversos profissionais. Barreiras incluíram as limitações das questões dos formulários, lentidão e instabilidade nos sistemas, sobrecarga dos trabalhadores e falta de confiança nas respostas dos usuários.

**Quadro 2:** Categorias e subcategorias emergentes das análises das respostas dos questionários eletrônicos, relacionadas aos facilitadores e barreiras na utilização dos formulários de marcadores do consumo alimentar na atenção primária à saúde.

CATEGORIAS	SUBCATEGORIAS	
	FACILITADORES	BARREIRAS
Estrutura do formulário	Múltipla escolha Rápido preenchimento Fácil entendimento	Questionário incompleto Ser referente ao dia anterior
Utilização do sistema	Informatização Interface simples	Estar em abas diferentes no sistema * Lentidão/Instabilidade do sistema Não migração de dados entre sistemas Problemas na identificação do usuário
Rotina do serviço de saúde	Aplicação por qualquer profissional	Falta de tempo e profissionais Sobrecarga de trabalho Adesão dos profissionais Falta de qualificação e sensibilização dos profissionais Falta de infraestrutura
Interação com o usuário	-	Falta de confiança nos usuários Resistência em responder às questões Insegurança alimentar e nutricional

Fonte: elaboração própria.

* Em 2023 os formulários de marcadores do consumo alimentar foram incluídos no Prontuário Eletrônico do Cidadão (PEC).

O Quadro 3 apresenta códigos emergentes dos grupos focais. Foram realizados 10 grupos focais, com 2 a 8 participantes cada (taxas de comparecimento entre 7% e 27%). As questões levantadas no questionário se refletiram nos grupos focais, com compreensão mais profunda.

**Quadro 3:** Códigos emergentes da análise de conteúdo dos grupos focais relacionados a facilitadores, barreiras e estratégias na utilização dos marcadores do consumo alimentar na atenção primária à saúde (APS).

TEMAS	CÓDIGOS	DESCRIÇÃO
Facilitadores	Infraestrutura adequada	Disponibilidade de recursos físicos adequados para utilização dos marcadores, incluindo salas, impressoras e informatização das UBS
	Estrutura dos marcadores	Objetividade, formato de múltipla escolha, e/ou estrutura dos marcadores, como formatação e informatização
	Aplicação por qualquer profissional	Compreensão e/ou importância do preenchimento dos marcadores do consumo alimentar não serem de responsabilidade exclusiva do nutricionista
	Facilidades das plataformas e-SUS APS e Sisvan Web	Interface e ferramentas das plataformas e-SUS APS e Sisvan Web, como geração de relatórios, identificação pelo número SUS e integração entre os sistemas
	Condicionalidades de políticas públicas	Engajamento na utilização dos marcadores do consumo alimentar devido à vinculação aos programas de políticas públicas e repasse financeiro às UBS
Barreiras	Falta de infraestrutura	Ausência de estrutura física e de material nos serviços, como indisponibilidade de salas, computadores insuficientes e internet instável, afetam negativamente o uso dos marcadores
	Limitações dos marcadores	Dificuldades enfrentadas no preenchimento e registro dos dados do consumo alimentar devido às questões serem restritas, referentes ao dia anterior, ou ainda quando o formulário é impresso
	Sensibilização dos profissionais	Falta de sensibilização, não compreensão da importância e não adesão ao uso dos marcadores e à coleta de dados por parte dos profissionais de saúde, incluindo nutricionistas
	Caracterização das plataformas de dados	Dificuldades no uso das plataformas eletrônicas, próprias ou não, devido à lentidão, à instabilidade, ou ainda por não estarem na mesma aba que o PEC *
	Situações da migração de dados	Demora na migração dos dados entre os sistemas (sistemas próprios, e-SUS APS e Sisvan Web) e desconfiança da fidedignidade e/ou perda dos dados
	Interação com o usuário	Dificuldade no entendimento das questões ou compreensão da importância dos marcadores e desconfiança da veracidade das respostas dos usuários
	Insegurança alimentar e seus casos	Constrangimento do usuário em responder os marcadores quando não há acesso regular e adequado à alimentos saudáveis ou ainda, quando o profissional sente-se desconfortável ante a situação
Estratégias	Ampliação da divulgação	Exemplos de divulgação dos dados para os próprios profissionais de saúde ou para a população de forma geral, por boletins, relatórios, reuniões, pesquisas científicas etc.
	Educação permanente como potência	Sensibilização, qualificação ou matriciamento, como rodas de conversa, oficinas e reuniões regulares, que abordem assuntos relacionados à alimentação e nutrição, como vigilância alimentar e nutricional ou o guia alimentar
	Ter apoio profissional para digitar os dados	Presença de profissionais para digitação dos dados nas plataformas, para diminuir o retrabalho dos profissionais de saúde da UBS
	Vai muito da forma de perguntar	Melhorias para a condução das perguntas dos marcadores, diminuindo o constrangimento e a omissão de respostas pelo usuário

PEC: Prontuário Eletrônico do Cidadão; SISVAN: Sistema de Vigilância Alimentar e Nutricional; SUS: Sistema Único de Saúde; UBS: unidade básica de saúde.

Fonte: elaboração própria.

* Em 2023 os formulários de marcadores do consumo alimentar foram incluídos no PEC.

A seguir, facilitadores e barreiras são apresentados conjuntamente, dada sua complementaridade. Estratégias discutidas entre participantes são delineadas sequencialmente, com potencial para melhorar a utilização dos marcadores. Os códigos estão destacados (itálico) e, quando relevante, acompanhados da categoria dos cargos assistenciais e de gestão que mais mencionaram o tema. Falas originais dos participantes exemplificam os códigos, identificados por profissão, macrorregião, cargo e sexo.

### Facilitadores e barreiras

Na percepção dos profissionais de cargos assistenciais, o uso dos marcadores do consumo alimentar é favorecido por *infraestrutura adequada*, incluindo disponibilidade suficiente de computador, impressora e toner e internet estável. Notaram ainda que unidades construídas recentemente e que recebem doações de equipamentos por parte de empresas do setor privado parecem ter melhor infraestrutura.

“*Eu acho que a vantagem e a parte boa é que em todas as UBS* [unidade básica de saúde] *a gente tem computador e internet, isso facilita muito o atendimento e é uma forma da gente fazer os marcadores, porque antigamente não tinha, era bem pior ou tinham poucos computadores*” (nutricionista, Sudeste, cargo de assistência, mulher).

Profissionais e gestores frequentemente mencionaram a *falta de infraestrutura*, destacando a escassez de espaço físico para atendimento, de computadores nas salas e de *tablets* para agentes comunitários de saúde (ACS) em visitas domiciliares. O acesso instável à internet e à energia elétrica também foi apontado. Para contornar a falta de equipamentos, alguns profissionais levam seus próprios computadores para a unidade de saúde. Quando não há computador disponível, optam por não usar os marcadores, pois sabem que não terão tempo para inserir os dados no sistema. Em algumas unidades, a falta de internet por mais de um dia leva ao acúmulo de dados para lançamento nos sistemas de informação. Prioriza-se, assim, o registro de atendimentos quando a conexão é restabelecida, em comparação à digitação dos marcadores.

Para UBS que adotam formulários impressos, os profissionais consideraram uma melhoria na *estrutura dos marcadores* a concentração das questões em uma única página, economizando recursos. No entanto relataram dificuldades na impressão devido à falta de papel e tinta. Além disso, os impressos podem resultar em retrabalho e perda de dados. Por isso, os gestores apontaram a informatização dos marcadores como uma facilidade para sua utilização.

“*A própria questão de imprimir os formulários para fazer fora do atendimento na unidade de saúde. Às vezes, não tem folha suficiente, não tem cartucho suficiente. O próprio computador, aqui não tinha uma sala para nutricionista, então tinha que contar com a disponibilidade dos computadores da unidade de saúde para poder registrar esses dados. Então, esses seriam os maiores obstáculos com relação aos marcadores*” (nutricionista, Sul, cargo de assistência, mulher).

A maioria dos profissionais da assistência considerou os marcadores do consumo alimentar, seja no formato impresso ou eletrônico, práticos e de preenchimento rápido, devido à abordagem concisa. Ressaltaram a compreensão fácil por parte dos usuários e trabalhadores das UBS. No entanto, alguns nutricionistas apontaram *limitações dos marcadores*, argumentando que questões fechadas e referentes ao dia anterior não permitem uma avaliação adequada dos hábitos alimentares.

Os gestores enfatizaram que a compreensão da possibilidade de *aplicação por qualquer profissional* facilita o uso dos marcadores nos serviços de saúde, ressaltando a necessidade de qualificação. Nesse sentido, a falta de *sensibilização dos profissionais* sobre a importância dos marcadores foi mencionada. Há uma percepção equivocada de que os marcadores são de responsabilidade exclusiva de nutricionistas, sem reconhecimento de seu potencial para diagnóstico do perfil alimentar da população, planejamento e avaliação de intervenções.

“*Os profissionais têm dificuldade de compreender a importância da ferramenta e muitas vezes eles atribuem como mais uma tarefa. Então, na minha unidade, por exemplo, eu disponibilizei para os ACS, para os enfermeiros e para todos os médicos. Tentei explicar a importância da ferramenta, mas infelizmente os profissionais da atenção básica, talvez não todos, mas a grande maioria, ainda estão... Como eu posso falar? Impulsionados pelo modelo curativista. Então é naquela lógica: Não é patologia, então não tem importância. Então é muito na contramão do que a atenção básica prevê, do que é dentro dos princípios e diretrizes da Política Nacional da Atenção Básica*” (enfermeira, Sudeste, cargo de gestão, mulher).

Existem *facilidades das plataformas* como o acesso universal pelo e-SUS APS, a integração entre sistemas e o registro do usuário pelo número do cartão SUS, com diminuição da sobrecarga de trabalho. Gestores consideraram o Sisvan Web prático para geração de relatórios. Porém, na *caracterização das plataformas*, profissionais da assistência reportaram enfrentar dificuldades devido à necessidade de habilitação para acesso e a problemas de lentidão no Sisvan Web. Muitos indicaram preferência pelo e-SUS APS por ser mais ágil.

“*Que quando você tira um relatório mais quantitativo, porque o e-SUS te traz um relatório mais quantitativo. E o do SISVAN é uma coisa linda, é a coisa mais linda do mundo porque ele traz aquela coisa de qualidade, o que ele estiver ele está te dando e oportunizando e que o e-SUS não traz isso para você*” (nutricionista, Nordeste, cargo de gestão, mulher).

Ademais, muitos profissionais e gestores relataram demora na *migração de dados*, prejudicando a representação da situação alimentar atual nos relatórios. A utilização de sistemas municipais próprios, a não obrigatoriedade do número do cartão de saúde no e-SUS APS e a desconfiança da migração de dados entre sistemas causam desmotivação no registro dos marcadores do consumo alimentar. Apesar da lentidão, alguns preferem inserir os dados via Sisvan Web para evitar perdas.

“*Analista de sistema sempre sinaliza para a gente que tem uns campos que têm de ser preenchidos para poder ter a migração. Se não for preenchido, não ocorre. Então há perda na informação e muita queixa em relação a isso. Quando a gente mostra o relatório: ‘o seu município está com tanto de informação’; ‘não, mas a gente fez muito mais’, mas se perdeu porque não preencheu os campos*” (nutricionista, Nordeste, cargo de gestão, mulher).

Profissionais da assistência indicaram desconfiar da veracidade das *respostas dos usuários*, ao notarem vergonha e omissão dos hábitos alimentares. Pode haver influência pela expectativa dos usuários quanto ao que os profissionais querem ouvir e resistência dos usuários ao uso dos marcadores por profissionais não nutricionistas. Desenhou-se um entendimento por maior confiabilidade às respostas com o autopreenchimento pelos usuários. Adicionalmente, foi referido que os usuários sentem constrangimento ao assumir a falta de alimentos em casa. Os participantes também apontaram desconforto quando se depararam com *casos de insegurança alimentar*, com hesitação para uso dos marcadores nestas circunstâncias.

“*Aqui no município, existe a expectativa de quem vai responder o questionário e de receber algo em troca a partir daquilo. É um município grande, vulnerável em seus dependentes, e muitas vezes o profissional também sente a dificuldade porque a pessoa nem sempre tem o que comer. Então quando você pergunta: ‘ontem você consumiu?’, é uma situação sem graça, a pessoa não teve o que comer, não teve acesso ao alimento. Então essas questões são bem frequentes aqui quando a gente reúne os profissionais, dessa insegurança alimentar*” (nutricionista, Sudeste, cargo de gestão, mulher).

Paralelamente, a *condicionalidade com políticas públicas* foi destacada como facilitadora, com menção à priorização do uso dos marcadores entre beneficiários do Programa Bolsa Família (PBF), à obrigatoriedade pelos Programas Saúde na Escola (PSE) e Crescer Saudável, à adesão das estratégias Amamenta e Alimenta Brasil (EAAB) e Prevenção e Atenção à Obesidade Infantil (Proteja), bem como ao repasse do Financiamento de Ações de Alimentação e Nutrição (FAN). A vinculação ao incentivo financeiro foi considerada eficiente por profissionais e gestores, pois foi percebida como um discurso bem aceito nos serviços de saúde.

“*Eu acho que uma das facilidades, um dos pontos altos que eu vejo é a permeabilidade que ele* [marcadores] *possui em distintos programas. Então, o Crescer Saudável, que comunica com a estratégia Amamenta Alimenta, que comunica com o PSE, que consegue dialogar dentro de uma sala de vacinação, ele é totalmente aplicado para os beneficiários do Programa Auxílio Brasil. Então, eu acho que não é uma ferramenta que coloca muros, mas que ela constrói pontes, porque ela acaba dialogando com inúmeras linhas de cuidados, de formas de fazer*” (enfermeira, Sudeste, cargo de gestão, mulher).

### Estratégias

Estratégias exitosas para incentivo do uso dos marcadores do consumo alimentar na rotina da APS foram compartilhadas pelos participantes dos grupos focais. A *ampliação da divulgação dos dados* de marcadores do consumo alimentar foi mencionada como uma estratégia importante para que todas as fases da vigilância alimentar e nutricional pudessem ser apreendidas, para além do preenchimento das informações, melhorando a compreensão da relevância dos marcadores. Entretanto, os profissionais da assistência narraram que é comum haver cobranças pela coleta de dados sem a devolutiva das análises para as unidades de saúde.

“*Então eu acho que tem um tanto não só uma sensibilização inicial, mas um reforço periódico com relação a importância, de dizer: ‘os dados diminuíram, os dados aumentaram’ e dar uma devolutiva com relação a isso. Acho que quando a gente aqui na base recebe essas devolutivas: ‘olha só que bacana, agora a gente consegue ver que dentro da sua unidade, dentro do seu território e dentro do seu município nós temos esse perfil de acordo com os dados que você preencheu’. Então essa devolutiva de ver o que está acontecendo com aquele papel que você preencheu ajuda o profissional a entender um pouco melhor e conseguir ver a importância, porque às vezes a gente só recebe pedidos de dados e a gente não recebe nada*” (nutricionista, Sudeste, cargo de assistência, mulher).

Meios de disseminação incluíram a criação de boletins mensais e a elaboração de relatórios por estagiários, com divulgação na UBS, secretarias de saúde, escolas participantes do PSE e conselhos de segurança alimentar. Universidades e institutos que utilizam dados do SISVAN para estudos também foram mencionados. Profissionais e gestores sugeriram a apresentação dos marcadores nos cursos de Ensino Superior, para que seus egressos cheguem familiarizados à ferramenta nos serviços.

Com relação à *educação permanente* dos profissionais, as qualificações nos equipamentos versam sobre a utilização dos marcadores com apoio de manuais já disponíveis sobre o SISVAN e as bases teóricas a partir dos guias alimentares, incluindo ACS, enfermeiros e educadores físicos. Foi relatada a importância de os profissionais saberem lidar com eventuais questões nutricionais, uma vez que as perguntas dos marcadores suscitam dúvidas sobre hábitos alimentares.

“*Eu fiz oficina com os ACS destacando uma parte do manual mesmo e de um guia que fala sobre o uso do marcador do consumo alimentar, eu destaquei as orientações para cada resposta de cada pergunta e dei a cada um dos ACS, para que eles diante da resposta até pudessem orientar aquela mãe para poderem justamente já irem entendendo o porquê não era só para eles pegarem os dados, que a intenção era de eles estarem fazendo educação alimentar e nutricional*” (nutricionista, Nordeste, cargo de assistência, mulher).

Os participantes apontaram a importância de treinamentos para digitadores para operarem os sistemas de maneira adequada, garantindo a confiabilidade dos dados. Ressalta-se que *ter apoio profissional para digitação dos dados* foi uma estratégia assinalada pelos profissionais da assistência para diminuir a sobrecarga de trabalho, mesmo quando o papel de digitador é assumido apenas quando há acúmulo de fichas.

Ainda, de acordo com gestores, a qualificação adequada para uso dos marcadores não permite lacunas que possam resultar em desconforto para o usuário. Foi pontuado que *vai muito da forma de perguntar*, ou seja, a abordagem do cuidado, influencia em como as perguntas são recebidas. Estratégias incluem não falar em tom de julgamento e praticar escuta qualificada e acolhimento, evitar iniciar consultas com os marcadores para não intimidar, e utilizá-los apenas quando sua importância for justificada.

“*Eu entendo que se a pessoa que está perguntando tiver sido minimamente orientada, inclusive de como conduzir, porque se a gente olhar o questionário, ele inclusivamente menciona ontem, ontem comeu. E aí eu sempre alerto para se a pessoa dizer: ‘Eu sempre como’, não é sempre, ‘Raramente como’, não é raramente. A gente sempre quer saber ontem. Eu acho que se a pessoa que está aplicando tiver sido minimamente orientada, não dá para nem gerar nenhum tipo de constrangimento, não*” (nutricionista, Norte, cargo de gestão, mulher).

“*Vai muito da forma de perguntar. Eu acho que a gente não começa uma consulta logo já perguntando: ‘o que foi que você comeu ontem?’, a gente vai perguntando ao longo do tempo. A gente inicia tipo assim, a gente não faz a pergunta de forma com que cause algum constrangimento. Já que a pessoa foi procurar um nutricionista, por exemplo, a gente diz que a gente precisa fazer algumas perguntas sobre hábito alimentar e a pessoa vai respondendo ao longo do tempo, aí a gente não percebe causar esse constrangimento*” (nutricionista, Nordeste, cargo de gestão, homem).

Finalmente, a Tabela 2 oferece a quantificação dos códigos originados nos grupos focais, de acordo com as categorias profissionais. Como facilitadores, profissionais e gestores destacaram, respectivamente, a estrutura dos marcadores e a aplicação por qualquer profissional e demarcaram a condicionalidade de políticas públicas. Barreiras percebidas entre profissionais e gestores foram entraves nas plataformas de dados e falta de sensibilização para uso dos marcadores. Entre as estratégias, gestores assinalaram particularmente a educação permanente e profissionais indicaram o apoio à digitação e formas de abordagem com usuários.

**Tabela 2:** Trechos codificados na análise de conteúdo temática estratificados por profissionais da assistência e gestores da atenção primária à saúde (APS).

Códigos	Profissionais da assistência	Profissionais da gestão
Facilitadores		
Infraestrutura adequada	8	2
Estrutura dos formulários	21	12
Aplicação por qualquer profissional	3	10
Facilidades das plataformas e-SUS APS e Sisvan Web	5	6
Condicionalidade de políticas públicas	13	13
Barreiras		
Falta de infraestrutura	12	10
Limitações do formulário	7	3
Sensibilização dos profissionais	14	14
Caracterização das plataformas de dados	18	26
Situações da migração de dados	7	9
Interação com o usuário	5	3
Insegurança alimentar e seus casos	6	7
Estratégias		
Ampliação da divulgação	7	10
Educação permanente como potência	5	16
Ter apoio profissional para digitar os dados	4	1
Vai muito da forma de perguntar	6	5

SISVAN: Sistema de Vigilância Alimentar e Nutricional; SUS: Sistema Único de Saúde.

Fonte: elaboração própria.

## Discussão

Este estudo qualitativo investigou, de forma inovadora, facilitadores, barreiras e estratégias para ampliar o uso dos marcadores do consumo alimentar na APS, com base nas experiências e interações entre profissionais e gestores, de todas as regiões brasileiras. O delineamento metodológico em duas camadas proporcionou aprofundamento dos temas, e promoveu a troca de estratégias entre os participantes. Profissionais da assistência destacaram a importância da infraestrutura, mas enfrentaram desafios por falta de recursos. Apesar da praticidade, alguns nutricionistas apontaram limitações aos marcadores. Gestores enfatizaram a necessidade de reconhecer que a ferramenta pode ser usada por diversos profissionais, não apenas nutricionistas. Estratégias incluíram a ampliação da divulgação dos dados dos marcadores e educação permanente, com suporte de documentos técnicos e embasamento teórico nos guias alimentares.

A crescente preocupação no cenário brasileiro com a morbimortalidade associada a fatores alimentares ^20^ ^,^ ^21^ salienta a necessidade de monitoramento adequado dos hábitos alimentares da população. Os relatórios do SISVAN disponibilizam diferentes estratificações, com grande potencial de apoio ao planejamento de programas e à reorganização da atenção nutricional em contextos locais ^22^. Evidências apontam que a sustentabilidade de um sistema de vigilância nutricional depende da demanda por informações, custo razoável, rápida geração e disseminação de informações de boa qualidade, integração às estruturas governamentais e uma instituição central organizadora ^23^. Entretanto, a baixa cobertura populacional dos marcadores ^9^ ainda compromete o alcance do SISVAN e a literatura é escassa em relação a estudos sobre as potencialidades do uso dessa ferramenta.

Observou-se que a maioria dos facilitadores e barreiras mencionados foram complementares entre si. Isso evidencia uma interdependência reconhecida pelos próprios profissionais: quando há condições favoráveis para o uso dos marcadores (facilitadores), como a condicionalidade com políticas públicas, a incorporação das atividades de vigilância alimentar e nutricional na prática cotidiana e a presença do nutricionista na equipe, sua utilização ocorre de forma mais fluida. Por outro lado, o potencial dos marcadores não é plenamente aproveitado quando os recursos são limitados (barreiras), a exemplo de dificuldades na utilização do sistema e a intensa rotina dos serviços de saúde, bem como falta de motivação, de qualificação e de infraestrutura. As estratégias, por sua vez, trouxeram experiências exitosas compartilhadas entre os participantes, profissionais e gestores, e que podem aprimorar a utilização dos marcadores.

Ações de vigilância alimentar e nutricional estão historicamente associadas a políticas públicas ^24^, o que pode ampliar coberturas populacionais, especialmente entre gestantes, crianças e adolescentes ^9^ ^,^ ^25^. Nos três ciclos de avaliação do Programa Nacional de Melhoria do Acesso e da Qualidade da Atenção Básica (PMAQ) foi reportada a institucionalização do PSE em todas as regiões do país, associado a maiores níveis de cobertura da Estratégia Saúde da Família (ESF) ^26^. Uma revisão da literatura científica destacou, dentre outros desfechos positivos, a relação entre o PBF e o aumento no acesso aos serviços de APS entre 2003 e 2020 ^27^. Esses achados ressaltam o impacto positivo das políticas públicas na cobertura da APS para garantir direitos à saúde e à alimentação adequada, incentivando o uso dos marcadores.

O nutricionista tem papel central para desenvolvimento das ações de alimentação e nutrição e a contratação desses profissionais para integrar equipes, alinhada às demandas locais, é recomendada por especialistas no fortalecimento da atenção nutricional na APS ^28^. No entanto, alguns nutricionistas apontaram que os marcadores podem não garantir a avaliação adequada dos hábitos alimentares. Deve-se notar que eles são uma ferramenta de triagem, projetada para avaliar rapidamente a alimentação, em formato simples e compatível para facilitar o uso na rotina da assistência ^8^. Assim, os marcadores facultam avaliar a qualidade de aspectos centrais da dieta ^6^ ^,^ ^7^, sem almejar empenhar uma análise de sua composição nutricional. É fundamental, portanto, que os profissionais estejam apropriados dessas características ao usar a ferramenta ^8^.

Além disso, a APS tem caráter interprofissional ^29^. Processos de qualificação interprofissional têm potencial para melhorar a colaboração nas equipes, sendo sua garantia de responsabilidade compartilhada entre profissionais e gestores ^24^ ^,^ ^30^. Assim, é importante que os marcadores do consumo alimentar sejam introduzidos ainda durante os cursos de graduação em nutrição e em outras áreas da saúde, como parte do currículo acadêmico, contribuindo para o conteúdo da temática de vigilância em saúde.

A publicação de materiais educativos e manuais técnicos ^8^ ^,^ ^31^ também pode apoiar a atitude de vigilância alimentar e nutricional ^22^, uma vez que tais documentos contribuem para a apreensão da finalidade e das etapas relacionadas a essas ações. Essa perspectiva geral sobre as atividades de vigilância alimentar e nutricional, especialmente em relação ao uso dos marcadores, é crucial para a implementação dessas práticas. No entanto deve ser reconhecida a preponderância de uma perspectiva gerencial, o que por vezes conflita com as práticas profissionais na assistência, conforme evidenciado em alguns códigos deste estudo. Rolim et al. ^10^, por exemplo, destacaram que a maioria dos responsáveis pelo SISVAN concentram suas atividades na coleta e digitação de dados, e as qualificações recebidas também priorizam essas etapas.

Profissionais e gestores mencionaram que, ainda que a coleta de dados condicionada a repasses financeiros impulsione o uso dos marcadores, também pode restringir a vigilância alimentar e nutricional a certos ciclos etários e focar no cumprimento de metas. Idealmente, o uso dos marcadores deve integrar o cuidado e ser parte de políticas públicas mais amplas, além do desenvolvimento de ações estratégicas em alimentação e nutrição nas UBS e o monitoramento de indicadores de vigilância alimentar e nutricional, incluindo o consumo de alimentos saudáveis e não saudáveis, com consequente incremento da cobertura populacional no país ^22^.

A divulgação dos dados locais pode ser um primeiro passo para envolvimento de profissionais e gestores com informações em nível do território, indo ao encontro da diretriz de descentralização do SUS. Devolutivas foram apontadas como estratégia interessante para sensibilização dos profissionais, sendo sugerida a elaboração de materiais como relatórios, mapas e boletins em termos acessíveis, para profissionais de saúde de diversas áreas, gestores, pessoas da sociedade civil e outros atores ^22^ ^,^ ^32^.

Ressalta-se que o encontro entre profissionais e gestores, conforme facilitado nesta pesquisa, pode oportunizar a fluidez do ciclo de gestão e produção do cuidado no exercício da vigilância alimentar e nutricional, permitindo que informações e ações sejam transmitidas e realizadas, tanto no nível individual quanto no coletivo. O ciclo de gestão vincula dados individuais em alimentação e nutrição aos sistemas de informação, gerando diagnósticos coletivos para embasar decisões e implementar estratégias ou políticas em níveis municipal, estadual ou federal ^31^. Conexões entre os atores envolvidos são essenciais para garantir um ciclo coeso, considerando os espaços de cuidado e os instrumentos necessários, com aprendizados derivados das práticas diárias ^24^ ^,^ ^30^.

Ainda, o valor dos marcadores aumenta à medida que são incorporados à assistência à saúde. Iniciativas muito relevantes nesta direção incluem os protocolos para orientações alimentares individuais a partir das recomendações dos guias alimentares para a população em geral ^33^. Esses protocolos conferem apoio ao raciocínio clínico e à abordagem do cuidado dos profissionais no que tange às possíveis questões alimentares dos usuários arrolando sugestões de condutas embasadas nas respostas aos marcadores de forma personalizada. Dessa forma, há consequência à avaliação dos marcadores do consumo alimentar, com sentido iminente à prática profissional nos serviços e repercussões positivas ao diagnóstico coletivo.

A relevância de abordagens acolhedoras foi mencionada, a partir de escuta qualificada, como uma forma de evitar constrangimentos ao acessar o consumo alimentar. Em comparação a métodos de avaliação com detalhamento extenso da dieta, os marcadores podem oferecer, inclusive, um canal mais sutil e gradual para compreensão de práticas alimentares, contribuindo para uma relação mais humanizada entre profissionais e usuários. Ademais, essa ferramenta pode auxiliar na identificação de situações de violação do direito humano à alimentação adequada especialmente se aliada ao emprego da Triagem para Risco de Insegurança Alimentar (TRIA) na APS ^31^.

Algumas limitações devem ser elucidadas sobre este estudo. Recomendações para a condução de grupos focais versam sobre um mínimo de quatro participantes ^34^, mas foram notadas dificuldades para comparecimento dos convidados e três sessões deste estudo foram realizadas com dois profissionais. Ainda, a participação com a câmera aberta não foi compulsória, levando participantes a optarem apenas pelo áudio, sem possibilidade de captura de expressões faciais. Em todos os casos, contudo, resguardou-se a fluidez da conversa entre os participantes, sendo os grupos mantidos para as análises. Essas dificuldades precisam ser ponderadas com as características e a demanda de trabalho dos serviços da APS, à luz dos desafios inerentes aos necessários movimentos de pesquisa com estes atores. Ademais, os critérios de saturação foram adequadamente atendidos.

Entre pontos fortes, destaca-se a abordagem metodológica com grupos focais, que se configuraram como espaço para trocas mútuas de experiências de profissionais e gestores que compartilham das mesmas dificuldades em seus cenários de prática. Entende-se que o uso de entrevistas semiestruturadas individuais, por exemplo, incorreria em limitações. Os grupos focais proporcionaram alguns pontos de reinvenção dos participantes, com movimentos de acolhimento e incentivo do uso dos marcadores.

Em suma, os resultados ampliam a compreensão dos desafios no uso dos marcadores do consumo alimentar na APS e ressaltam a importância da vinculação a políticas de repasses de recursos e da participação de profissionais de diversas áreas nas ações de vigilância alimentar e nutricional. Conclui-se que há necessidade de maiores investimentos em infraestrutura, qualificação profissional e divulgação dos dados dos marcadores do consumo alimentar, fortalecendo o ciclo de gestão e produção do cuidado. Além disso, a análise das barreiras pode estimular a colaboração entre equipes da APS, enfatizando a troca de estratégias entre diferentes contextos, visando fortalecer o monitoramento do consumo alimentar no SUS.
